# Progress in Vaccine-Preventable and Respiratory Infectious Diseases—First 10 Years of the CDC National Center for Immunization and Respiratory Diseases, 2006–2015

**DOI:** 10.3201/eid2407.171699

**Published:** 2018-07

**Authors:** Anne Schuchat, Larry J. Anderson, Lance E. Rodewald, Nancy J. Cox, Rana Hajjeh, Mark A. Pallansch, Nancy E. Messonnier, Daniel B. Jernigan, Melinda Wharton

**Affiliations:** Centers for Disease Control and Prevention, Atlanta, Georgia, USA

**Keywords:** Centers for Disease Control and Prevention, CDC, National Center for Immunization and Respiratory Diseases, NCIRD, immunization, respiratory infections, respiratory infectious diseases, vaccine-preventable diseases, vaccines, pneumonia, influenza, influenza virus, viruses, polio, Haemophilus influenzae type b, Hib, bacteria

## Abstract

The need for closer linkages between scientific and programmatic areas focused on addressing vaccine-preventable and acute respiratory infections led to establishment of the National Center for Immunization and Respiratory Diseases (NCIRD) at the Centers for Disease Control and Prevention. During its first 10 years (2006–2015), NCIRD worked with partners to improve preparedness and response to pandemic influenza and other emergent respiratory infections, provide an evidence base for addition of 7 newly recommended vaccines, and modernize vaccine distribution. Clinical tools were developed for improved conversations with parents, which helped sustain childhood immunization as a social norm. Coverage increased for vaccines to protect adolescents against pertussis, meningococcal meningitis, and human papillomavirus–associated cancers. NCIRD programs supported outbreak response for new respiratory pathogens and oversaw response of the Centers for Disease Control and Prevention to the 2009 influenza A(H1N1) pandemic. Other national public health institutes might also find closer linkages between epidemiology, laboratory, and immunization programs useful.

By 2005, global spread of highly pathogenic avian influenza A(H5N1) (*1*), major disruption of US vaccine supplies (*2*), and anticipated introduction of multiple new vaccines, including those targeting emerging drug-resistant respiratory infections, provided a rationale for the Centers for Disease Control and Prevention (CDC) to establish the National Center for Immunization and Respiratory Diseases (NCIRD). A closer linkage between science and program was an explicit goal of the center’s formation, and NCIRD has been on the cutting edge of applying advances in technology to public health. In April 2006, the new center brought scientific units responsible for epidemiologic and laboratory aspects of most vaccine-preventable and other acute respiratory infectious diseases together with programs supporting public sector immunization. The center’s activities aligned to address 8 initial strategic priorities (Table 1).

**Table 1:** Strategic priorities for the National Center for Immunization and Respiratory Diseases, Centers for Disease Control and Prevention

Strategic priorities	Implementation examples
Improve immunization programs	Implemented central vaccine distribution and Vaccine Tracking System (VtrkS); supported development of adolescent platform for vaccination
Strengthen systems to evaluate policy effectiveness	Initiated annual estimation of influenza vaccine impact based on influenza surveillance, vaccine effectiveness studies, and immunization coverage surveys; introduced National Immunization Survey-Teen and quality standards for systems monitoring school-based immunization coverage
Accelerate vaccine-preventable disease reduction worldwide	Implementing partner for the Hib Initiative (2005–2009), which facilitated decisions to introduce *Haemophilus influenzae* b (Hib)–containing vaccine in all Global Alliance for Vaccines and Immunization–eligible countries and provided model framework for subsequent new vaccine introduction efforts
Reduce complications of pneumonia and influenza	Issued evidence-based guidance for influenza antiviral use to reduce severity of influenza illness
Improve pandemic preparedness	Enhanced laboratory detection of novel influenza viruses and led response to first influenza pandemic of the 21st century
Strengthen response to respiratory outbreaks	Developed Unexplained Respiratory Disease Outbreak tool kit for state, local, and international partners (https://www.cdc.gov/urdo/index.html)
Develop and promote strategies to reduce respiratory infections, vaccine-preventable diseases, and control antimicrobial resistance	Expanded the Advisory Committee on Immunization Practices recommendations for annual influenza vaccination and age groups recommended for pneumococcal conjugate vaccination; incorporated Get Smart: “know when antibiotics work” activities into national strategy to reduce antimicrobial resistance
Improve identification of causes of respiratory infections	Validated TaqMan technology for multiple pathogen diagnosis of respiratory syndromes; completed multicenter studies of etiology of pneumonia in the community in adults and children

NCIRD concentrated the expertise of the agency on the viral and bacterial agents that cause pneumonia, influenza, and other acute respiratory syndromes; responsibilities for tuberculosis remained elsewhere (Technical Appendix). The expanded list of newly vaccine-preventable diseases meant response in areas traditionally managed by communicable disease units relied on immunization expertise, and the newer vaccines in turn required enhanced laboratory-based surveillance for accurate postlicensure evaluations. The mission of NCIRD was to prevent disease, disability, and death through immunization and control of respiratory and related infectious diseases. The center embedded field staff within and provided funding and technical assistance to state, local, and territorial health departments to strengthen detection, prevention, and control of these conditions, with particular emphasis on childhood immunization, influenza, and emerging respiratory infectious disease threats. The center also housed several World Health Organization (WHO) International Collaborating Centers and provided leadership for global laboratory networks for influenza, polio, measles, rotavirus, and bacterial meningitis, among others.

During the subsequent decade, NCIRD established strong evaluation systems to measure policy and program impacts. NCIRD also spearheaded modernization of the nation’s immunization activities and collaborated with other infectious disease programs to invest in advanced molecular detection technology to accelerate prevention, detection, and control of influenza and other respiratory threats. The center aimed to sustain the public’s acceptance of vaccination while providing technical assistance and on-the-ground support for outbreak responses to previously rare vaccine-preventable diseases. In 2009, the staff of NCIRD led the response of CDC to the first influenza pandemic of the 21st century. In subsequent years, response of NCIRD to other novel respiratory threats, such as Middle East respiratory syndrome coronavirus, severe respiratory illness associated with enterovirus D68, and increases in Legionnaires’ disease, involved collaboration with clinicians, state, and local health departments and, when appropriate, international organizations and ministries of health.

## Immunization System Change at CDC and Health Departments

**Figure 1:**
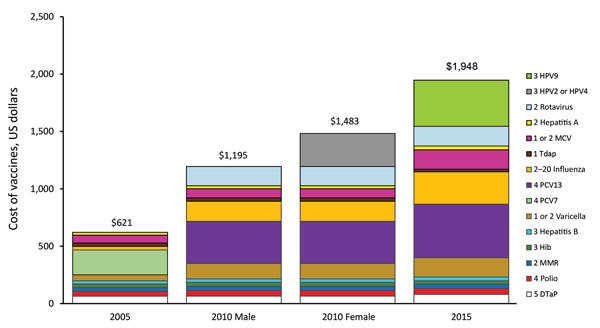
Cost of vaccines routinely recommended from birth through 18 years of age, according to public sector contract prices, United States, 2005, 2010 (by sex), and 2015. Values in key indicate no. vaccinations/series. Data were based on federal contract prices as of September 1, 2005; April 6, 2010; and April 1, 2015. DTaP, diphtheria, tetanus, and acellular pertussis vaccine; Hib, *Haemophilus influenzae* type b vaccine; HPV, human papillomavirus vaccine; MCV, meningococcal conjugate vaccine; MMR, measles, mumps, and rubella vaccine, PCV, pneumococcal conjugate vaccine; Tdap, tetanus, diphtheria, and acellular pertussis vaccine.

**Figure 2:**
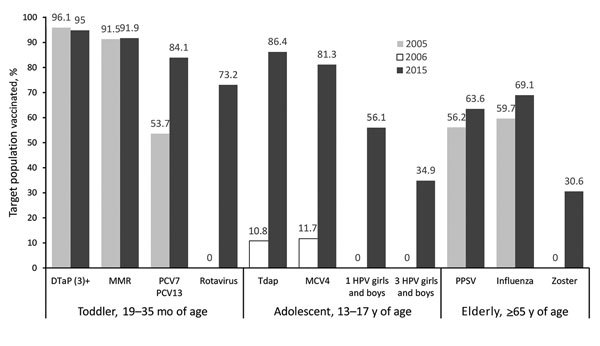
Selected immunization coverage by vaccine and target group, United States, 2005–2006 and 2015. PCV7 and PCV13 are >4 doses. Rotavirus coverage is 2 or 3 doses depending on product. PCV7 is a pneumococcal conjugate vaccine with 7 serotypes, and PCV13 is a pneumococcal conjugate vaccine with 13 serotypes. Data were obtained from the National Immunization Survey for toddlers, the National Immunization Survey-Teen (NIS-Teen) for adolescents, the National Immunization Survey-Flu for influenza, and the National Health Interview Survey for PPSV and zoster. DTaP, diphtheria, tetanus, and acellular pertussis vaccine; HPV, human papillomavirus vaccine; MCV, meningococcal conjugate vaccine; MMR, measles, mumps, and rubella vaccine; PCV, pneumococcal conjugate vaccine; PPSV, pneumococcal polysaccharide vaccine; Tdap, tetanus, diphtheria, and acellular pertussis vaccine.

During 2005–2015, the childhood immunization schedule expanded substantially, with corresponding increased costs (Figure 1), sustained high immunization coverage of traditional vaccines, and increased coverage of newer vaccines (Figure 2). Licensure of several vaccines and shifting epidemiology in selected vaccine-preventable diseases placed a premium on strong surveillance and evaluation systems to provide evidence needed for policy. The Advisory Committee on Immunization Practices (ACIP) released numerous recommendations during this period (Table 2). Clinicians developed an adolescent platform to permit delivery of vaccines to children 11–12 years of age. The program introduced the National Immunization Survey-Teen (a large telephone survey of parents, with healthcare provider verification of records) in 2006 to monitor coverage of immunizations in persons 13–17 years of age. Expanded monitoring of influenza vaccine coverage relied on several new techniques, including leveraging hospital reporting to the Center for Medicare and Medicaid Services, which incentivized reporting of influenza vaccination of healthcare workers.

**Table 2:** Selected recommendations of the Advisory Committee on Immunization Practices, Centers for Disease Control and Prevention, 2005–2015*

New vaccine (indication)	Date of ACIP recommendation
Meningococcal ACWY conjugate (adolescents)	2005 Feb
Tdap (adolescents)	2005 Jun
Measles, mumps, rubella, and varicella (MMRV)	2005 Oct
Influenza (24–59-month-old children)	2006 Feb
Rotavirus (infants)	2006 Feb
Human papillomavirus (adolescent girls)	2006 Jun
Second-dose varicella	2006 Jun
Zoster (shingles)	2006 Oct
Influenza (persons 6 mo–18 y of age)	2009 Feb
13-valent pneumococcal conjugate vaccine (children)	2010 Feb
Influenza (universal in persons >6 mo of age)	2010 Feb
Second dose of meningococcal ACWY conjugate (adolescents)	2010 Oct
Human papillomavirus (adolescent boys)	2011 Oct
Tdap (in every pregnant woman)	2012 Oct
13-valent pneumococcal conjugate vaccine (persons >65 years of age)	2014 Aug
Meningococcal B vaccine (during outbreaks and in high-risk persons)	2015 Feb
HPV-9 (adolescents)	2015 Feb
Meningococcal B (adolescents, category B)	2015 Jun

*ACIP, Advisory Committee on Immunization Practices; HPV, human papillomavirus; Tdap, tetanus, diphtheria, and acellular pertussis.

Monitoring of vaccine performance resulted in updates to vaccine recommendations. Continued varicella outbreaks led to recommendation of a second dose of varicella-containing vaccine. Emergence of waning immunity following meningococcal conjugate vaccine (Men ACWY) prompted recommendation for a booster dose. Resurgence of pertussis prompted investigations of vaccine performance and characterization of circulating strains. Surveillance detected emergence of pertactin-deficient *Bordetella pertussis* and epidemiologic studies documented the limited duration of protection afforded by acellular vaccines (*3*). Accordingly, updated tetanus, diphtheria, and acellular pertussis vaccine recommendations advised women to receive this vaccine during each pregnancy to prevent severe disease in early infancy.

NCIRD implemented changes that modernized the nation’s immunization infrastructure and strengthened the program’s efficiency. Establishment of a centralized vaccine distribution system in 2008 eliminated 99% of the 430 vaccine depots that states previously relied on to store vaccines, resulting in direct shipping to >40,000 provider sites, with reduced inventory costs and waste. NCIRD replaced a DOS-based vaccine ordering and management system with the vaccine tracking system VtrkS (https://www.cdc.gov/vaccines/programs/vtrcks/index.html), an SAP-enabled data management and analytic system (https://www.sap.com/products/data-services.html), thereby improving the accountability of the CDC multibillion-dollar vaccine supply chain. Improvements in immunization information systems (i.e., registries) have been slowed by diversity of registries in states; barriers to interstate data sharing; and incomplete records, particularly for adult vaccinations. Investments focused on increasing their interoperability with electronic medical records, expanding physician use, developing clinical decision support tools (e.g., prompts for which vaccine doses are due or overdue), and improving efficiency and accuracy of data entry.

Provisions of the Affordable Care Act of 2010 regarding prevention services required updated insurance plans to include all vaccines routinely recommended by the ACIP without copayments when administered by an in-network provider. The Vaccines for Children Program, implemented in 1994, provides free vaccines for children who are either uninsured or Medicaid eligible, among others (http://www.cdc.gov/vaccines/programs/vfc/index.html). By requiring first dollar coverage for ACIP recommended vaccines, the Affordable Care Act provision eliminated most out-of-pocket costs for persons with either public or private insurance. A notable exception occurs for vaccines covered under Medicare part D (e.g., shingles), for which senior citizens might still have large out-of-pocket expenditures. NCIRD helped health departments establish billing systems so that insurance companies paid for vaccines given to their covered members, saving limited public sector resources (https://www.cdc.gov/vaccines/programs/billables-project/success-stories.html).

Protecting the infrastructure supporting immunization was essential during the decade, even once most insurance plans fully covered vaccination (*4*). State, local, and federal immunization programs responded to resurgent pertussis; outbreaks of measles, mumps, and meningococcal meningitis; extended shortages of *Haemophilus influenzae* type b (Hib) conjugate, and 5-in-1 infant combination vaccines. Enhancements made at the national level included randomly sampling cellular telephones and land lines to access participants for the National Immunization Survey (*5*), and strengthening the link between pediatric immunization caregivers, oncologists, and cancer survivor advocates in an effort to improve human papillomavirus vaccination of children 11 and 12 years of age (https://www.cancer.org/health-care-professionals/national-hpv-vaccination-roundtable.html).

Sustaining high levels of vaccine acceptance was also a focus. Based on formative research with parents and clinicians, and in partnership with the American Academy of Pediatrics, NCIRD released a communication tool kit aimed at promoting effective vaccine conversations between providers and parents. CDC enhanced transparency of the ACIP process by introducing Grading of Recommendations Assessment, Development and Evaluation for systematic review of the evidence (*6*), and webcast the committee’s public meetings (*7*). Although vaccinating children remained a social norm during this period, with <1% of toddlers receiving no vaccine doses (*8*), outbreaks of measles identified geographic areas where personal belief exemptions were becoming more common (*9*,*10*).

On the occasion of the 20th anniversary of implementation of the Vaccines for Children program, CDC staff completed analyses of the impact of childhood immunization. Twenty years of childhood immunization, at actual coverage levels, averted 322 million illnesses and 732,000 premature deaths, at a net savings of $295 billion in direct costs and $1.38 trillion in total societal costs (*11*). The childhood immunization series was estimated to save $3 in direct medical costs for each dollar invested (*12*).

## Improving Prevention and Control of Respiratory Infections

Substantial improvements in control of respiratory infections in the United States occurred during the decade. Scientists in industry and academia have advanced molecular detection for respiratory pathogens during this period, and NCIRD staff capitalized on advancements to apply tools to the public health need for detection of respiratory infections (*13*), thereby extending their value beyond commercial or clinically relevant applications. NCIRD applied new multipathogen diagnostic tests to characterize the burden of community-acquired pneumonia in the United States (*13*–*15*) and collaborated on similar efforts in developing country settings (*16*,*17*).

**Figure 3:**
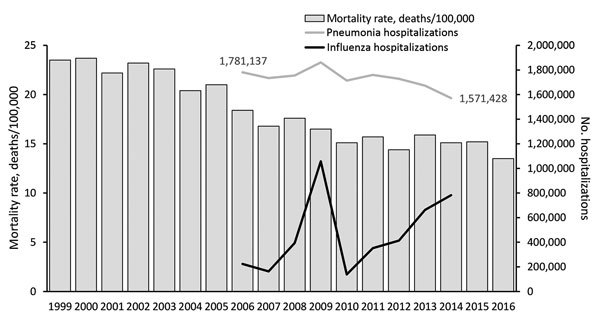
Pneumonia and influenza mortality rates and hospitalization counts by year, United States, 1999–2016. Age-adjusted mortality rates were obtained from the National Center for Health Statistics and based on underlying cause of death; nationwide hospitalization estimates were obtained from the Agency for Healthcare Research and Quality Healthcare Cost and Utilization Project.

Hospitalizations and deaths from respiratory infectious disease in the United States continued to decrease during this period. Pneumonia and influenza decreased from the sixth to eighth leading cause of death in the United States during 2006–2015, and age-adjusted mortality rates decreased by 17.4%, from 18.4 deaths/100,000 persons in 2006 to 15.2 deaths/100,000 persons in 2015 (*18*). Severe influenza seasons contributed to variability in influenza hospitalizations, but pneumonia hospitalizations decreased by 11.7%, from 1,781,137 in 2006 to 1,571,428 in 2014 (Figure 3).

Several advances probably contributed to the decrease in all-cause pneumonia hospitalizations. Pediatric use of pneumococcal conjugate vaccine (PCV) averted an estimated 47,000 (43%) hospitalizations in children <2 years of age and 168,000 pneumonia hospitalizations in all age groups by 7–9 years after introduction of 7-valent PCV (PCV7) vaccine (*19*). Although increases in drug-resistant nonvaccine serotype invasive pneumococcal disease occurred, an expanded serotype formulation, a 13-valent pneumococcal conjugate vaccine (PCV13), replaced PCV7 in 2010. The Active Bacterial Core surveillance of the Emerging Infections Program Network conducted studies of serotype-specific changes in invasive pneumococcal disease during the decade, which suggest that much of the decrease in adult pneumococcal illness was caused by reduced transmission of vaccine-type pneumococci from vaccinated children to adults (*20*). In addition, highly active antiretroviral therapy reduced the extremely high risk for pneumonia among persons infected with HIV and likely lowered all-cause pneumonia burden in this population (*21*).

Given the higher risk for invasive pneumococcal disease associated with active and passive cigarette smoke, reductions in cigarette smoking among adults and in secondhand smoke exposure during this period might also have reduced pneumonia (*22*–*24*). Annual vaccination rates against seasonal influenza among adults >65 years of age increased from 59.7% in 2005 to 69.1% in 2015 (*25*), and vaccination rates among other age groups, especially children, increased substantially. In August 2014, PCV13 was recommended for persons >65 years of age, in addition to the longstanding recommendation for 23-valent pneumococcal polysaccharide vaccine (*26*). Quantifying the independent role any of these factors played in the decrease in hospitalizations and deaths would be difficult.

During this period, program monitoring focused on strengthening the link between data and action. NCIRD collaborations sped up the availability of data on influenza vaccine coverage and effectiveness. Interim estimates of early vaccine coverage were released each December for the ongoing influenza season, and preliminary estimates of the effectiveness of the influenza vaccine for that season were disseminated during the first quarter of each calendar year (*27*). Final data from each season on influenza illness, vaccine effectiveness, and vaccine coverage were incorporated into a model that estimated the annual impact of vaccination and the incremental benefit that would be achieved with increased coverage of current vaccines or with future vaccines with higher efficacy (*28*,*29*). The model estimates that, from 2010–11 through 2015–16, a total of 26,808,150 illnesses and 415,115 hospitalizations were prevented through seasonal influenza vaccination (*30*). Influenza vaccination was associated with reduced risk for laboratory-confirmed influenza-associated pediatric death (*31*).

In 2004–05, state and local public health and clinicians faced substantial challenges from a sudden decrease in expected influenza vaccine doses for the US market (*2*). Investments of the US government and vaccine manufacturers led to a diversified influenza vaccine supply and substantially more doses produced in more recent years. New formulations include quadrivalent, cell-based, recombinant, high-dose, and adjuvanted products (*32*). Universal recommendations for annual vaccination and more predictable supply resulted in more persons in the United States immunized against influenza. Data from the National Health Interview Survey suggest the proportion of all US adults receiving influenza vaccine increased from 27.4% in 2005–06 (*33*) to 44.8% in 2014–15 (*34*).

Annual measurements of vaccine effectiveness show major limitations in protection by current vaccines. New questions concern possible lower effectiveness after sequential vaccination and lower effectiveness against influenza A(H3N2) virus. Public health practitioners and clinicians thus face new challenges, despite ample supply, promoting the imperfect vaccines currently available. Although researchers focus on development of universal influenza vaccines, incremental improvements, such as cell-derived vaccine candidate viruses, which can avoid egg-adapted vaccine virus changes, offer promise in the interim.

## Strengthening Health Protection and Response

In conjunction with national strategies for pandemic preparedness issued by the US government (*35*,*36*), CDC worked with state and local health departments, clinicians, nongovernmental organizations, and global partners to strengthen readiness for avian and pandemic influenza. Exercises helped ready public health authorities for pandemics and other biologic threats, including assessment of community mitigation strategies. Public engagement efforts conducted in 2005–2009 permitted planners to incorporate values of citizens into prioritization for scarce vaccine supplies and led to development of strategies to reach workers in critical infrastructure fields (*37*). CDC supported strengthened detection (e.g., enhanced diagnostic and surveillance systems) and improved response capabilities for influenza (*38*,*39*). Investments and technical assistance also focused on upgrading capacities within state and local public health agencies in the United States and ministries of health in other countries to address other emerging or severe respiratory pathogens.

On April 21, 2009, CDC reported 2 human cases of respiratory illness in southern California caused by a novel influenza virus that had a previously unseen combination of genes that originated from viruses circulating previously in pigs, birds, and humans (*40*,*41*). Additional cases in California, Texas, New York, and Mexico led to recognition of the 2009 influenza A(H1N1) pandemic. Elderly populations had some cross-protection against the virus, but children and younger adults were disproportionately affected (*42*). Facilitated by preparedness investments, the influenza laboratory of NCIRD was able to adapt a PCR test for detection of the pandemic H1N1 virus, obtain Emergency Use Authorization from the US Food and Drug Administration, and rapidly ship new diagnostic test kits to states and 153 countries (*38*). CDC and its partners rapidly prepared a candidate vaccine virus so that the multiple steps of vaccine manufacturing could get under way quickly. The pandemic response effort of CDC supported epidemiologic and virologic investigations, adapted the centralized vaccine distribution system used for the Vaccines for Children Program, and made 330,000 shipments of monovalent influenza vaccine. CDC worked with state and local health departments to support vaccination at high-throughput public clinics, private provider offices, occupational clinics and, eventually, pharmacies. During October–December 2009, more than 80 million persons in the United States received the monovalent H1N1 vaccine. The pandemic response incorporated risk communication strategies, frequent media briefs, and messages shared through trusted community-based partners to reach diverse populations. The initial response included large numbers of school closures, which were highly disruptive. Updated policy advised more limited use of school dismissals.

Unfortunately, large amounts of vaccine were not available until several weeks after the peak of the fall pandemic wave. The 2009 H1N1 pandemic virus caused lower overall severe illness in the fall of 2009 than had been expected. However, in 2014–15, circulation of a drifted influenza A(H3N2) virus caused substantially more severe disease, resulting in an estimated 707,155 hospitalizations (*28*,*30*).

During its first decade, NCIRD also addressed several novel respiratory pathogens, including enterovirus D68 and concurrent increases in acute flaccid myelitis in children. Emergence of virulent avian influenza A(H7N9) virus in China in 2013 prompted intensified surveillance and prepandemic vaccine investments. Evolution of viruses and geographic spread has further increased concern (*43*). During 2012–2015, sporadic cases and outbreaks of severe respiratory illness caused by the newly recognized Middle East respiratory syndrome coronavirus were detected in Saudi Arabia and elsewhere on the Arabian Peninsula. As occurred for severe acute respiratory syndrome coronavirus and Ebola virus, transmission in hospital settings affected patients and healthcare workers (*44*). NCIRD coordinated agency preparedness and response to Middle East respiratory syndrome. When the virus was imported to Indiana and Florida in the United States by 2 healthcare workers who had been exposed in hospitals in Saudi Arabia, prompt detection and response included effective infection control and contact tracing. No further spread from these importations occurred.

## Global Vaccine-Preventable Disease Progress

Vaccine-preventable disease control has benefited from longstanding efforts by the United Nations Children’s Fund, the WHO Expanded Program on Immunization, and the Global Polio Eradication Initiative, among others. NCIRD collaborated with these and newer organizations, such as the Global Alliance for Vaccines and Immunization (Gavi), which supports new and underused vaccination in low-resource countries.

**Figure 4:**
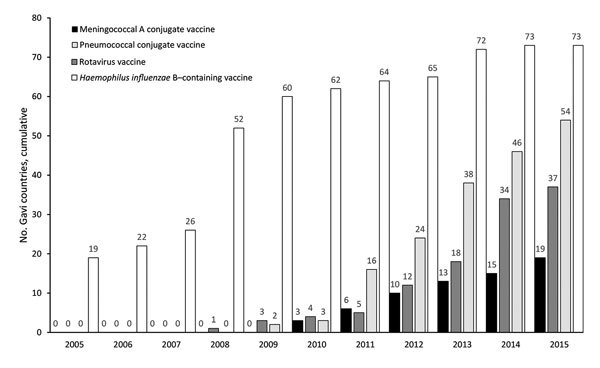
Cumulative number of Gavi-eligible countries using selected new or underused vaccines in routine programs, 2005–2015. Data were obtained from Gavi. Gavi, Global Alliance for Vaccines and Immunization.

The first decade of NCIRD coincided with major progress in accelerated uptake of new or underused vaccines in developing countries. NCIRD epidemiologic and laboratory studies contributed to the evidence base in support of broader use of pneumococcal conjugate and rotavirus vaccines, and NCIRD staff played key leadership or technical roles in public–private partnerships, such as the Hib Initiative (*45*) and pneumococcal and rotavirus Accelerated Development and Introduction Plans (*46*), funded by Gavi, and provided laboratory and statistical support to the Meningitis Vaccine Project led by PATH (Seattle, WA, USA) and WHO. Use of Hib vaccine increased from 19 Gavi-supported countries in 2005 to all 73 by 2014. Use of pneumococcal conjugate increased to 54 countries, and use of rotavirus vaccines increased to 37 countries (Figure 4). MenAfriVac, developed by Serum Institute of India with support from the Meningitis Vaccine Project, was administered in campaigns during 2010–2015 that reached 235 million persons within the African meningitis belt. These efforts have eliminated epidemics of group A meningococcal meningitis (*47*), although more recent outbreaks of group C meningococcal disease have occurred. During this period, NCIRD assisted ministries of health in assessing benefits of the newer vaccines (*46*,*48*,*49*), as well as the occurrence and favorable benefit-risk ratio associated with intussusception after rotavirus vaccination (*46*).

Global eradication of polio has been a priority since 1988. During 2006–2015, polio transmission was interrupted in Egypt and India, and rapid response efforts halted outbreaks of imported polio in many counties in Africa, as well as in Tajikistan and the Middle East. By 2015, wild poliovirus continued to circulate in Afghanistan, Pakistan, and Nigeria, although annual case counts had decreased from 1,997 wild poliovirus cases in 2006 to 74 in 2015. NCIRD scientists led the Global Polio Eradication Initiative laboratory network, enhanced approaches to environmental monitoring, and assessed use of bivalent and monovalent oral polio vaccine and fractional doses of inactivated polio vaccine as tools for achieving eradication. In 2011, the Global Immunization Division moved from NCIRD to the newly formed Center for Global Health to continue programmatic aspects of eradication and vaccine-preventable disease control. Laboratories at CDC for vaccine-preventable diseases, including polio and measles, remained in NCIRD. Strong collaboration between the Global Immunization Division and NCIRD has continued, as has sharing of the US experience with vaccine hesitancy with partners facing similar challenges in other regions.

## Ongoing Challenges

Globalization and microbial adaptation continue to threaten disease control progress. Sustaining community acceptance is needed even once effective and safe interventions are available. Although technological improvements to laboratory detection, vaccine development and production, and information systems have been marked during the first decade of NCIRD, application to public health, including immunization and disease reporting, is slow. Business drivers to improvements in electronic health records and fragmentation of the public health enterprise (e.g., different immunization registries in each state) have limited progress. Another challenge has been lower than expected vaccine performance (e.g., acellular pertussis, live attenuated influenza vaccine). Development of influenza vaccines with broader protection is a priority, but their availability remains years away. Decreasing survey response rates threaten continuity of health monitoring, while the potential of big data for conditions requiring precise laboratory confirmation, such as respiratory pathogens, might be limited. The Global Health Security Agenda includes roadmaps and accountability metrics to strengthen response to emerging threats, but sustained governmental and private sector commitments to this effort will be essential.

## Conclusions

Establishment of a center housing epidemiologic, laboratory, and program units related to vaccine-preventable and acute respiratory infectious disease provided a platform for strategic improvements to disease prevention and control that was timely given the influenza pandemic in 2009, the changing epidemiology of vaccine-preventable diseases, and increased demands on the immunization system in the United States. NCIRD worked in concert with state and local public health, as well as with numerous global partners, to optimize use of available prevention tools, strengthen detection and control measures, and build the evidence base for the next generation of interventions, such as improved influenza vaccines and vaccines in development against respiratory syncytial virus. Pandemic influenza and Ebola showed that implementing vaccination or testing new countermeasures in the midst of epidemics is difficult. Global initiatives suggest that partnerships between public and private sectors, engaging scientific and programmatic perspectives, might achieve more impact than single-agency efforts.

As ministries of health in industrialized and resource-poor countries increase attention to pandemic-prone respiratory infections and expand their immunization programs to address common endemic infectious diseases, organizational change similar to ours might be beneficial. However, organizational design cannot address or anticipate every issue, and will not eliminate the interdependence of programs or the value of collaboration across programs, sectors, and nations.

Technical Appendix Additional information on progress in vaccine-preventable and respiratory infectious diseases—the first 10 years of the CDC National Center for Immunization and Respiratory Diseases, 2006–2015.
