# Supplementary material for: Progress in Vaccine-Preventable and Respiratory Infectious Diseases—First 10 Years of the CDC National Center for Immunization and Respiratory Diseases, 2006–2015
Source: Emerg Infect Dis. 2018 Jul;24(7):1178–87. doi: 10.3201/eid2407.171699 (PMC6038744; doi:10.3201/eid2407.171699)
Supplement: Technical Appendix — Additional information on progress in vaccine-preventable and respiratory infectious diseases—the first 10 years of the CDC National Center for Immunization and Respiratory Diseases, 2006–2015. [file 17-1699-Techapp-s1.pdf]

Article DOI: https://doi.org/10.3201/eid2407.171699

Progress in Vaccine-Preventable and Respiratory Infectious Diseases—First 10 Years of the CDC National Center for Immunization and Respiratory Diseases, 2006–2015

Technical Appendix

Organizational Roots of the National Center for Immunization and Respiratory Diseases

Rationale for Change

Increasing public health attention to avian influenza and pandemic preparedness, complexity of the scientific response needs for outbreaks of severe respiratory disease of unknown etiology (e.g., severe acute respiratory syndrome [SARS]), and interdependence of influenza scientists and immunization program staff of the Centers for Disease Control and Prevention (CDC), as exemplified by response to the sudden 2004 influenza vaccine supply shortage, suggested the value of a center that would specialize in acute respiratory infectious diseases and link scientific and programmatic efforts. Emergency supplemental funding for influenza led to rapid growth of the Influenza Branch and need for a larger infrastructure to support new extramural and international programs. In addition, several new vaccines were being added to routine immunization schedules, resulting in a shift in priorities for state and local public health immunization programs. The expense of new vaccines prompted need for improved vaccine management. Some new vaccines targeted common nonspecific clinical syndromes (e.g., pneumonia, gastroenteritis) that relied heavily on laboratory confirmation to monitor vaccine effectiveness and program performance. Expertise related to the newer vaccines (e.g., for pneumococcus, meningococcus, rotavirus, shingles) had been housed in the National Center for Infectious Diseases. However, the National Immunization Program had no laboratories but enjoyed a large field staff embedded in state and local public health.

Organizational Predecessors

Components of the National Center for Infectious Diseases (NCID) at CDC and the entire National Immunization Program (NIP) came together in forming the National Center for Immunization and Respiratory Diseases (NCIRD), which initially included 5 divisions and an Office of the Director housing centralized communication, information technology, and policy assets. Components of the Division of Bacterial and Mycotic Diseases and the Division of Viral and Rickettsial Diseases of NCID, which conducted epidemiologic, laboratory, statistical, and communication activities related to acute respiratory infections, joined staff from the National Immunization Programs Epidemiology and Surveillance Division in formation of new divisions within NCIRD: the Division of Bacterial Diseases, the Division of Viral Diseases, and the Influenza Division. The other 2 divisions of NIP, the Immunization Services Division and the Global Immunization Division, initially remained unchanged within the new center's structure.

Other Respiratory Infectious Diseases

Tuberculosis activities are located in multiple components of CDC. The Division of TB Prevention and Control focuses on control of tuberculosis in the United States, includes epidemiology, laboratory, program support to state and local health departments, and clinical trial expertise, and is housed in the National Center for HIV/AIDS, Viral Hepatitis, STD, and TB Prevention. This center includes other control programs that rely on contact tracing and case-management of chronic infections. Additional tuberculosis activities occur in the Division of Global Migration and Quarantine, within the National Center for Emerging and Zoonotic Infectious Diseases, where the quarantine authority and health issues related to refugees, immigrants, and visitors are a focus. International tuberculosis activities occur within the Division of Global HIV/AIDS and Tuberculosis within the Center for Global Health. The clinical presentation for tuberculosis usually differs from that for acute pneumonia, bronchiolitis, and meningitis, and treatment recommendations are quite distinct. Public health response to individual cases is essential for tuberculosis, whereas public health typically intervenes for outbreak control and surveillance purposes for acute respiratory infections.

Other Causes of Acute Respiratory Infections

Although most acute respiratory infectious diseases are addressed by components within NCIRD, those diseases caused by fungi (e.g., histoplasmosis, coccidioidomycosis) are the responsibility of the Division of Foodborne, Waterborne, and Enteric Diseases, and those spread

by vectors (e.g., hantavirus pulmonary disease, pulmonic plague) are the responsibility of the Division of Vector-Borne Infectious Diseases, both in the National Center for Emerging and Zoonotic Infectious Diseases. CDC experts from across these units work together on enhancing diagnostics for multiple pathogens for respiratory syndromes, support state and local health departments for investigations of respiratory outbreaks (http://www.cdc.gov/urdo), and triage management of outbreaks of respiratory diseases of unknown etiology. Multiple centers also collaborate on waterborne infectious diseases, including Legionnaires disease, for which control relies on strengthening water management systems in buildings.

Relevance for Ministries of Health and State Health Departments

No organizational structure is perfect. Technical and financial resources and national priorities might address emerging program needs through temporary management changes (e.g., a yearlong SARS Task Force was formed at CDC to implement lessons learned after the SARS epidemic) or long-term organizational change, as was implemented with formation of NCIRD. Some state health departments found a compelling rationale to reorganize components after the influenza A(H1N1) pandemic in 2009, suggested by need for closer collaboration between preparedness and immunization units based on response experience. Although state and local health departments are a key constituent for the US CDC, healthcare organizations are also major constituents for NCIRD, because respiratory infections are a leading cause of outpatient visits and hospitalizations, and represent the eighth leading cause of death in the United States. State health departments are less likely to need a structure that separates respiratory infections from other communicable diseases, but some National Public Health Institutes might derive value from following the model of NCIRD, building on their influenza activities to investigate other respiratory infections and tightening the linkages between respiratory disease control and immunization programs.
